# Does Platelet-Rich Plasma Freeze-Thawing Influence Growth Factor Release and Their Effects on Chondrocytes and Synoviocytes?

**DOI:** 10.1155/2014/692913

**Published:** 2014-07-17

**Authors:** Alice Roffi, Giuseppe Filardo, Elisa Assirelli, Carola Cavallo, Annarita Cenacchi, Andrea Facchini, Brunella Grigolo, Elizaveta Kon, Erminia Mariani, Loredana Pratelli, Lia Pulsatelli, Maurilio Marcacci

**Affiliations:** ^1^Nano-Biotechnology Laboratory, Rizzoli Orthopaedic Institute, Via di Barbiano 1, 40136 Bologna, Italy; ^2^II Clinic-Biomechanics Laboratory and Nano-Biotechnology Laboratory, Rizzoli Orthopaedic Institute, Via di Barbiano 1/10, 40136 Bologna, Italy; ^3^Laboratory of Immunorheumatology and Tissue Regeneration/RAMSES, Rizzoli Orthopaedic Institute, Via di Barbiano 1/10, 40136 Bologna, Italy; ^4^Laboratory RAMSES, Rizzoli Orthopaedic Institute, Via di Barbiano 1/10, 40136 Bologna, Italy; ^5^Immunohematology and Transfusion Medicine and Cell and Musculoskeletal Tissue Bank, Rizzoli Orthopaedic Institute, Via di Barbiano 1/10, 40136 Bologna, Italy; ^6^Laboratory of Immunorheumatology and Tissue Regeneration Rizzoli Orthopaedic Institute, Via di Barbiano 1/10, 40136 Bologna, Italy; ^7^Department of Medical and Surgical Science, University of Bologna, Via Giuseppe Massarenti 9, 40138 Bologna, Italy; ^8^Clinical Pathology Unit, Rizzoli Orthopaedic Institute, Via di Barbiano 1/10, 40136 Bologna, Italy

## Abstract

PRP cryopreservation remains a controversial point. Our purpose was to investigate the effect of freezing/thawing on PRP molecule release, and its effects on the metabolism of chondrocytes and synoviocytes. PRP was prepared from 10 volunteers, and a half volume underwent one freezing/thawing cycle. IL-1*β*, HGF, PDGF AB/BB, TGF-*β*1, and VEGF were assayed 1 hour and 7 days after activation. Culture media of chondrocytes and synoviocytes were supplemented with fresh or frozen PRP, and, at 7 days, proliferation, gene expression, and secreted proteins levels were evaluated. Results showed that in the freeze-thawed PRP the immediate and delayed molecule releases were similar or slightly lower than those in fresh PRP. TGF-*β*1 and PDGF AB/BB concentrations were significantly reduced after freezing both at 1 hour and at 7 days, whereas HGF concentration was significantly lower in frozen PRP at 7 days. In fresh PRP IL-1*β* and HGF concentrations underwent a significant further increase after 7 days. Similar gene expression was found in chondrocytes cultured with both PRPs, whereas in synoviocytes HGF gene expression was higher in frozen PRP. PRP cryopreservation is a safe procedure, which sufficiently preserves PRP quality and its ability to induce proliferation and the production of ECM components in chondrocytes and synoviocytes.

## 1. Introduction

The use of platelet concentrates is becoming very popular in the field of musculoskeletal tissue regeneration. A widespread interest has been shown for platelet-rich plasma (PRP) as an injective treatment or as a surgical augmentation procedure for the repair of tissues with low healing potential, with an increasing number of preclinical and clinical studies over time [1–6]. The rationale behind the use of this kind of treatment is the release, from platelets *α*-granules, of bioactive molecules such as growth factors (GFs) which play important roles in the regulation of growth and development of several tissues [7]. These molecules bind to the transmembrane receptors of their target cells regulating cell signaling pathways [1, 7, 8]. Alpha-granules also contain cytokines, chemokines, and many other bioactive proteins that stimulate chemotaxis, cell proliferation, and maturation, modulate inflammatory molecules, and attract leukocytes [8].

Preclinical evidence supports the use of intra-articular PRP injections for joint degenerative pathology, by targeting not only cartilage but also synovial and meniscal tissues and thus promoting a favorable environment for joint tissue healing [1]. However, few high-quality trials have been published, which show the clinical usefulness of PRP but only with an improvement limited over time and mainly in younger patients not affected by advanced degeneration [1, 9–11]. Although overall preclinical results are positive, clinical findings are less exciting and sometimes controversial. This can be explained at least in part by the difficulties in the clinical research in this field, which arise from the lack of standardized protocols both to produce and administer PRP, thus resulting in many variables that might influence the clinical outcome [12].

Platelet count, activation methods, leukocytes/red blood cells content, and number of injections are the most debated aspects that led to a different effect on target tissues. PRP storage is also a key factor: freeze-thawing allows easier patient management, but it is thought to impair platelet function and lifespan, alter the GF release pattern, favor the accumulation of pyrogenic cytokines, and increase the risk of bacterial proliferation [2]. For these reasons some authors prefer the fresh administration of PRP immediately after its preparation, thus requiring blood harvesting for each injection in case of multiple treatments [13]. Conversely, some researchers have used freeze/thaw cycles as an activation method of PRP in* in vitro* studies, since the effect of freeze/thawing is to physically damage the platelet membranes and therefore to initiate the release of the granule content. However, there is no consensus regarding the precise number of freeze/thaw cycles necessary for complete degranulation, which can vary from 2 to 4, or concerning the possibly negative effect on platelets and their bioactive molecules [13].

Whether freeze/thawing PRP might lead to different release kinetics of molecules or different effects on tissue homeostasis with respect to fresh PRP is poorly understood. Perut et al. [14] reported a reduction of platelet and leukocyte number without affecting the bFGF release in frozen PRP compared to fresh PRP. Moreover, proliferation and mineralization of bone marrow-derived mesenchymal stem cell culture were similar in both PRPs, thus suggesting that PG cryopreservation should be regarded as a safe procedure that does not affect the final properties of platelet concentrate and allows adequate quality control of the product [14]. Conversely, other authors believe that freeze-thawing PRP may lead to deleterious changes in the product and therefore negatively affect the efficacy of platelet concentrate [15].

The purpose of this study was to compare fresh and frozen PRPs by analyzing whether the different state might influence the release of bioactive molecules and their effects on chondrocytes and synoviocytes. In particular, since some authors showed that 95% of these molecules are secreted within 1 hour after activation and then platelets release additional proteins in one week [16], we tested whether there was any difference in GFs release after 1 h (immediate release) and 7 days for both PRPs. Moreover, we investigated whether freeze/thawing might affect the biological activity of PRP on chondrocyte and synoviocyte cultures.

## 2. Materials and Methods

The study was approved by the Institutional Review Board and the local Ethics Committee, and written informed consent was signed by each donor.

Ten healthy volunteers (Caucasian male, age range: 27–38 years, BMI: normal values) were enrolled to undergo a blood sample collection. Exclusion criteria were systemic disorders, infections, smoking, nonsteroidal anti-inflammatory drug use 5 days before blood donation, haemoglobin values lower than 11 g/dL, and platelet values lower than 150 × 10^3^/*μ*L. Subject anonymity was assured by assigning a code to each sample.

### 2.1. PRP Preparations

To prepare PRP, a 150 mL venous blood sample was collected in a bag containing 21 mL of sodium citrate and centrifuged at 730 g for 15 min. Most of the red blood cells were eliminated and the resulting plasma and buffy-coat were transferred to a separate bag through a closed circuit. After a second centrifugation at 3800 g for 10 min, the supernatant was collected to produce PRP [14]. Platelet and white blood cell concentrations were determined by a hematology analyzer (COULTER LH 750): linearity was 5–1000 × 10^3^/*μ*L for the platelet count and 0.1–100 × 10^3^/*μ*L for the white blood cell count. A half-volume of the PRP obtained was frozen at −30°C for 2 h and then thawed in a dry thermostat at 37°C for 30 min just before activation (frozen PRP). Both PRPs (fresh and frozen) from each donor were tested on chondrocytes and synoviocytes, without pooling them.

### 2.2. Evaluation of Factors Released from PRP

All PRP samples (fresh and frozen) were activated with 10% CaCl_2_ (22.8 mM final concentration) and divided into two aliquots, one incubated for 1 h and the other for 7 days at 37°C in 5% CO_2_, in agreement with the cell culture scheduled time point and PRP clinical administration [9].

After centrifugation (for 15 min at 2800 g at 20°C), the released supernatant was collected and stored at −30°C. Interleukin (IL)-1*β*, hepatocyte growth factor (HGF), platelet-derived growth factor AB/BB (PDGFAB/BB), transforming growth factor *β*1 (TGF-*β*1), and vascular endothelial growth factor (VEGF) were assayed using commercially available bead-based sandwich immunoassay kits (Bio-Rad Laboratories, Hercules, CA, USA; and Millipore Corporation, Billerica, MA, USA) [20]. The immunocomplexes formed on distinct beads were quantified by using the Bio-Plex Protein Array System (Bio-Rad Laboratories). Data were analyzed by using the Bio-Plex Manager software version 6.0 (Bio-Rad Laboratories). Standard levels between 70% and 130% of the expected values were considered accurate and were used.

### 2.3. Cell Isolation and Culture

Chondrocytes (*n* = 4; male, age range 62–73 years) and synoviocytes (*n* = 3; male, age range 68–73 years) were isolated from patients with OA (Kellgren-Lawrence grades II-III [21]) undergoing joint surgery. Cells were isolated by enzymatic digestion. Briefly, cartilage and synovial tissues were washed twice in phosphate buffered saline (PBS) and minced into small pieces. Chondrocytes were isolated by an enzymatic procedure as previously described and used at passage 3 [22].

Synoviocytes were obtained from synovial tissue that was digested with 0.1% Trypsin (Sigma-Aldrich) in PBS at 37°C, 5% CO_2_ for 30 minutes, and subsequently with 0.1% collagenase P (Roche) at 37°C for 1 hour under constant rotation.

Chondrocytes were plated at a density of 0.25 × 10^5^ cells/cm^2^ in 12-well tissue-culture plates and cultured for 24 h in a humidified atmosphere at 37°C with 5% CO_2_ in Dulbecco Modified Eagle Medium (DMEM; Sigma, St. Louis, Missouri) without fetal bovine serum to permit them to adhere to the wells.

Synoviocytes were plated at a density of 0.20–0.25 × 10^5^ cells/cm^2^ in 12-well tissue-culture plates and maintained for 24 hours in serum free OPTIMEM (Gibco-BRL, Life Technologies Grand Island, NY, USA) culture medium supplemented with 100 U/mL penicillin, 100 Mg/mL streptomycin (Invitrogen, Carlsbad, CA, USA) in a humidified atmosphere at 37°C with 5% CO_2_. Then, culture media of both chondrocytes and synoviocytes were supplemented with either fresh PRP or frozen PRP at 10% (vol/vol) previously activated with 10% calcium chloride (CaCl_2 _22.8 mM final concentration) to produce a platelet gel and release the granule content. The incubation period was seven days, during which time the culture medium was not changed. To maintain PRP activated platelets in contact with chondrocyte and synoviocyte monolayers while avoiding direct mixing, a Transwell device was used (pore 0.4 *μ*m; Corning, Costar). All experiments were run in parallel.

At the end of the incubation time (7 days), culture supernatants were collected and maintained at −80°C until their use in ELISA tests, whereas chondrocytes and synoviocytes were used for proliferation assay and then lysed for RNA extraction.

### 2.4. Proliferation Assay

Chondrocyte and synoviocyte growth in the presence of each PRP formulation was evaluated through the Alamar blue test [23]. Briefly, the cells were incubated with 10% of Alamar Blue for 3 hours and the fluorescence was measured with use of a microplate reader (CytoFluor 2350, Millipore). The results were expressed as a percentage of Alamar Blue reduction as indicated by the manufacturer's data sheet (AbD Serotec, Oxford, United Kingdom).

### 2.5. Chondrocyte and Synoviocyte Gene Expression Analysis

The expression of specific genes by chondrocytes was assayed with real-time quantitative reverse transcriptase polymerase chain reaction (RT-PCR). IL-1*β*, IL-6, IL-8/CXCL8, tumor necrosis factor-*α* (TNF-*α*), IL-10, metalloproteinase-13 (MMP-13), tissue inhibitor of metalloproteinase (TIMP)-1, VEGF, TGF*β*1, FGF-2, HGF, hyaluronic acid (HA) synthases (HAS)-2, aggrecan, collagen II, and Sox-9 were determined. The expression of specific genes by synoviocytes was assayed with RT-PCR, and IL-1*β*, IL-6, IL-8/CXCL8, TNF-*α*, IL-10, IL-4, IL-13, MMP-13, TIMP-1, TIMP-3, TIMP-4, VEGF, TGF*β*1, FGF-2, HGF, HAS-1, HAS-2, and HAS-3 were analyzed. Total RNA was isolated using TRIZOL reagent (Invitrogen) following the manufacturer's recommended protocol. RNA was reverse-transcribed using SuperScript First-Strand kit (Invitrogen).

RNA specific primers for PCR amplification were generated from GeneBank sequences using Primer 3 software (Table 1). Real-time PCR was run on the LightCycler Instrument (Roche) using the SYBR Premix Ex Taq (TaKaRa biotechnology). The calculated RNA messenger (mRNA) levels for each target gene were normalized to glyceraldehyde-3 phosphate dehydrogenase (GAPDH, reference gene), according to the ΔΔCt method; the data were calculated as the ratio of each gene to GAPDH and expressed as “number of molecules per 100,000 GAPDH.”

### 2.6. Measurement of HA an Lubricin Levels

HA levels were measured in the supernatants of chondrocytes and synoviocytes that had been treated for 7 days with PRP and frozen PRP, using commercial DuoSet ELISA kit (R&D Systems) following the manufacturer's instructions. Lubricin protein level was measured in the supernatants of chondrocytes using a specific Elisa Kit (PRG4) (Uscn, Life Science Inc., Wuhan, China).

### 2.7. Statistical Analysis

Data concerning the characterization of the PRP and frozen PRP were analyzed by Friedman's test for multiple comparisons of paired data and, when significant, followed by Bonferroni's post hoc correction for multiple comparisons (a value of *P* < 0.0125 was considered significant after Bonferroni's correction). Results obtained from the evaluations of the biological activity of PRP on chondrocyte and synoviocyte cultures were analyzed by the Wilcoxon matched-pairs test for multiple comparisons.

Statistical analysis was carried out using the Statistica for Windows package release 6.1 (Statsoft Inc., Tulsa, OK) and GraphPad Prism for Windows (CA, USA).

## 3. Results

### 3.1. Characterization of PRP and Frozen PRP

Table 2 shows the characterization of both fresh/frozen PRPs used in the study. Platelet concentration in PRP presented a median value of 929,000/*μ*L (interquartile range 720,000–965,000) and was about 3-fold lower in frozen PRP. White blood cells (WBC) maintained concentrations that overlapped those of the peripheral blood in PRP (median 5,500/*μ*L; interquartile range 5,000–6,500) and underwent an evident decrease after freezing, being below the detection limit in seven subjects and about 3-fold lower than the starting values in the remaining ones.

The soluble factors analyzed showed that in frozen PRP both the immediate and delayed releases were similar or slightly lower than those of PRP. In particular, the TGF-*β*1 and PDGF AB/BB concentrations were significantly reduced after freezing both at 1 h and at 7 days (*P* < 0.01), whereas the HGF concentration was significantly lower in frozen PRP only after 7 days (*P* < 0.01). IL-1*β* and VEGF concentrations were not significantly modified after PRP freezing, whatever the time of incubation.

Concerning time-related modifications, in fresh PRP, IL-1*β* and HGF concentrations underwent a significant increase after 7 days (*P* < 0.01), whereas the other factors were unmodified by time in both PRPs.

### 3.2. Chondrocyte and Synoviocyte Proliferation Assay and Gene Expression Analysis

Chondrocytes and synoviocytes grown in the presence of 10% of both PRPs were viable and able to proliferate up to 7 days with no difference between preparations (data not shown). At 7 days, cells are subconfluent.

**Figure 1:**
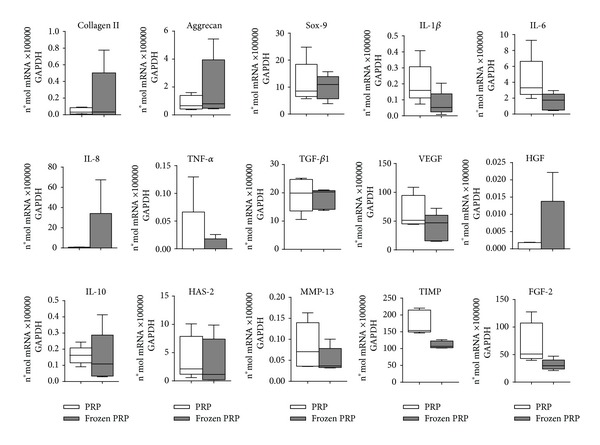
RT-PCR analysis: messenger RNAs expression in chondrocytes grown in presence of 10% fresh or frozen PRP at 7 days. Data were normalized to GAPDH and expressed as a percentage of the reference gene. Boxes indicate the 25% and 75% percentiles, whiskers indicate the minimum to maximum values, and bars indicate the median.

Collagen type II, aggrecan, and Sox-9 mRNAs were similarly expressed on day 7 in chondrocytes treated with fresh and frozen PRP (Figure 1). A different trend was observed for some of the inflammatory genes evaluated, in particular IL-1*β* and IL-6, which were highly induced by fresh PRP; however, these differences were not statistically significant (Figure 1). IL-8 and TNF-*α* were similarly expressed by the cells grown in presence of both fresh and frozen PRPs (Figure 1). No differences between fresh and frozen PRP were observed for TGF-*β*1, VEGF, HGF, IL-10, HAS-2, and MMP-13 expression. mRNAs for TIMP-1 and FGF-2 were highly induced by fresh PRP compared to frozen PRP, even if these values were not statistically significantly different.

**Figure 2:**
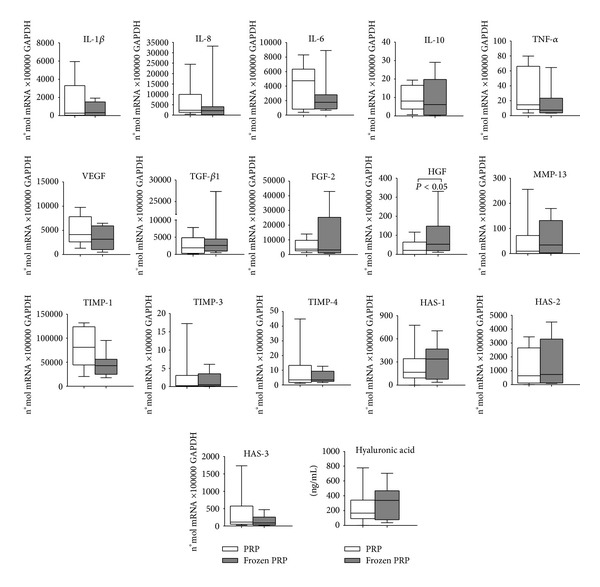
Expression analysis of factors involved in joint physiopathology: messenger RNAs expression in synoviocytes grown in presence of 10% fresh or frozen PRP at 7 days. Data were expressed as n°mol mRNA ×100000 GAPDH. Hyaluronic acid production was evaluated in culture supernatants and protein production was normalized per number of cells. Boxes indicate the 25% and 75% percentiles, whiskers indicate the minimum to maximum values, and bars indicate the median.

Gene expression analysis on synoviocyte cultures indicated that, among proinflammatory factors, anti-inflammatory factors, and/or anticatabolic factors, IL-1*β*, IL-8/CXCL8, IL-6, IL-10, and TNF-*α* were not differently modulated by the two preparations, whereas IL-4 and IL-13 gene expression levels were not detectable (Figure 2).

With regard to GFs, cartilage matrix degrading enzymes, and their inhibitors, no difference was found in synoviocyte gene expression levels between fresh PRP and frozen PRP for most of them (VEGF, TGF-*β*1, FGF-2, MMP-13, TIMP-1, TIMP-3, and TIMP-4). A significantly different gene expression level was found for HGF, which was higher in frozen PRP (*P* = 0.0313). HA synthases gene expression did not seem to be differently influenced by the two PRP preparations (Figures 1 and 2).

### 3.3. Hyaluronan Production

**Figure 3:**
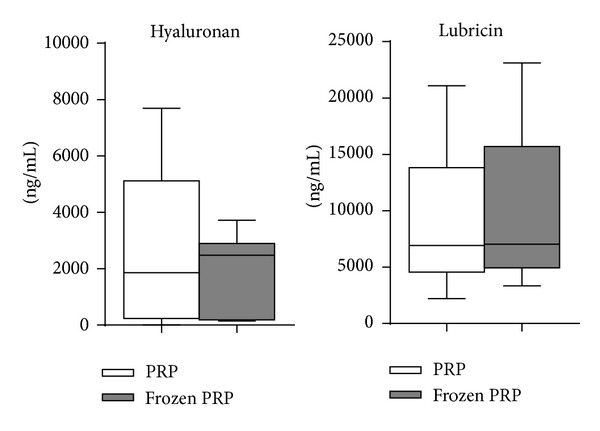
Hyaluronan levels in the culture media of human chondrocytes grown in presence of 10% fresh or frozen PRP at 7 days. Mean values are expressed as ng/mL, boxes indicate the 25% and 75% percentiles, whiskers indicate the minimum to maximum values, and bars indicate the median.

As shown in Figures 2 and 3, no differences were observed between fresh PRP and frozen PRP in hyaluronan secretion for both cell cultures and in lubricin production in chondrocytes.

## 4. Discussion

The heterogeneous clinical outcome reported in the literature on PRP treatment for joint tissue regeneration reflects the lack of guidelines regarding the use of platelet concentrates, starting from their production up to their clinical application. The increasing awareness on the need for PRP standardization is shown by the numerous biological studies investigating the role of each PRP variable on the healing potential of platelet concentrates [24–26]. Among these factors, the possibility to store PRP and the effects of freeze/thawing PRP remain a controversial point. Although some researchers avoid freeze/thawing, fearing deleterious effects on platelet function and GF release [2], others consider it to be a procedure that physically activates PRP [13].

In this scenario, our aim was to investigate whether PRP freezing/thawing affected the release of GFs from platelets *α*-granules at two key experimental points: 1 hour (immediate release) and 7 days, as scheduled delivery time in the clinical application, by evaluating the effects on chondrocyte and synoviocyte cultures, the main cell populations targeted in the joint.

The results of GF release showed that in frozen PRP the immediate release and the total amount at 7 days were not the same as in fresh PRP. Indeed, it seemed to be similar or slightly lower with respect to the fresh preparation, as reported for TGF-*β*1 and PDGF AB/BB. Similar results were reported by Durante et al. [27], who showed a slight downregulation of all PDGF isoforms after repeating cycles of freezing and thawing. Conversely, they also described an increased level of TGF-*β*1. No significant differences were detected for VEGF in this study. A different situation was described for HGF, which showed similar concentrations in both PRPs at 1 hour, but at 7 days a significant difference was observed with a higher amount of HGF in fresh PRP with respect to that of frozen PRP. One possible explanation might be linked to platelet storage-derived lesions: it has been reported [28] that during freezing storage platelets decrease in their responsiveness to aggregating agents and thus appear to reflect a general loss in the ability to become activated by various agonists. However, the functional defects of cold stored platelets do not appear to be as extensive as those of room temperature stored platelets [28]. Thus, freeze/thawing might still be a valid option to store PRP, although in this case frozen PRP might be less sensitive than fresh PRP to CaCl_2_, by not liberating the total amount of GFs stored in the *α*-granules, and some platelets and bioactive molecules might be damaged.

With regard to proinflammatory cytokines, the immediate release of IL-1*β* in this study did not significantly differ in fresh PRP with respect to that of frozen PRP at 1 hour and 7 days. Interestingly, even if not statistically different, there was a lower amount of IL-1*β* in frozen PRP with respect to that of fresh PRP (14.4 pg/mL versus 70.1 pg/mL, resp.), and a significant trend of a further increase of IL-1*β* from 1 hour to 7 days was observed only in fresh PRP. In a previous study [29] we observed that the IL-1*β* levels might be strongly correlated with WBC count. So, the absence of an increment of IL-1*β* detected in frozen PRP might be explained by the fact that freezing and thawing led to leukocyte destruction (which can produce inflammatory cytokines directly and indirectly by platelet stimulation), as we observed with the dramatic reduction in WBC number, preventing the “*de novo*” synthesis of this cytokine.

Another key point is the release kinetics of GFs in both PRPs. It has been recently reported that once platelets are activated, an initial burst of GF release is followed by a further sustained release, 3- to 5-fold increase as compared with baseline [30]. Our results highlighted a possible deviation from this GF release kinetics for frozen PRP, which showed no significant differences in proteins release at 1 h and 7 days, thus indicating a lower GF secretion over time. In fresh PRP samples, overall more bioactive molecules were released both at 1 hour and at 7 days, and both IL-1*β* and HGF release showed an increase at 7 days with respect to 1 hour.

Despite these differences in GF release, fresh and frozen PRPs did not differ in their ability to induce cell proliferation or ECM production and secretion in both chondrocytes and synoviocytes. Concerning gene expression analysis, chondrocytes cultured with both PRPs showed similar results for collagen II, aggrecan, and Sox-9, thus indicating that frozen PRP did not lose or reduce its ability to enhance chondrocyte anabolism. Albeit with no statistical significant difference with respect to frozen PRP, IL-1*β*, IL-6, FGF-2, and TIMP-1 were highly induced by fresh PRP. It could be speculated that their amount might be ascribed to leukocytes in fresh PRP. In fact, it has been reported that leukocytes may be responsible for the increased expression of IL-1*β*, IL-6, FGF-2, and TIMP-1 [31], and this might explain why their presence was not so marked in frozen PRP, in which freeze/thawing caused leukocyte destruction.

Concerning synoviocyte culture, also in this case the two PRP preparations did not induce significant differences in the expression of pro/anti-inflammatory agents and anticatabolic factors, whereas a higher expression of HGF was found in frozen PRP (*P* = 0.0313). Since it has been reported that IL-1*β* inhibits the synovial production of HGF [32] and since slightly lower levels of IL-1*β* are found in frozen PRP with respect to fresh PRP (which, however, was the only one to present a significant increase over time), the high level of HGF gene expression reached in synoviocytes incubated with frozen PRP might be ascribable to the reduction of the potential inhibitory effect of IL-1*β*. HGF has been shown to exert an anti-inflammatory effect on human chondrocytes, by downregulating nuclear factor kappa B, the main transcription factor involved in the inflammatory process [33]. Moreover, it has been reported that platelet activation increases levels of anti-inflammatory cytokines because of the presence of HGF [34]. Since there is increasing evidence that synovial inflammation plays a critical role in the symptoms and structural progression of OA [34], the level of HGF (which also exerts proangiogenetic effects) might be an intriguing aspect to be further explored in PRP procedures.

Current research aims to optimize PRP production and administration protocols. This study underlines two interesting aspects. The first one is that freeze/thawing affects PRP cell composition and its release of bioactive molecules. The second is that this different release kinetics does not significantly influence the effects on cell cultures. It is important to recognize that biological studies give important indications for the development of treatments, but their results do not always directly translate into clinical findings, as previously shown by the same clinical outcome reported using two biologically completely different procedures [35]. However, until clinical studies explore and clarify the effects of PRP storage on patient symptoms and functional improvement, this study suggests that freeze/thawing does not significantly affect PRP and can be considered as a storage option and thus simplify the management of patients undergoing multiple injection cycles of PRP.

## 5. Conclusion

PRP freezing is a controversial topic. Our results on GF release from platelets *α*-granules showed that in frozen PRP both the immediate and 7-day release were lower with respect to that of the fresh preparation, but without affecting the ability of PRP to induce proliferation and ECM production in chondrocyte and synoviocyte cultures. The only significant difference was detected for synoviocyte HGF expression, which was higher in the freeze/thawed PRP induced cells, thus suggesting that PRP cryopreservation is a safe procedure, which sufficiently preserves PRP quality and its biological activity.

## Figures and Tables

**Table 1:** List of primers used in real-time PCR.

RNA template	Primer sequences (5′–3′)	Annealing temperature (°C)	References∗
GAPDH	5′-TGGTATCGTGGAAGGACTCATGAC 3′-ATGCCAGTGAGCTTCCCGTTCAGC	60	[17]

Collagen type II	5′-GACAATCTGGCTCCCAAC 3′-ACAGTCTTGCCCCACTTAC	60	PRIMER 3

Aggrecan	5′-TCGAGGACAGCGAGGCC 3′-TCGAGGGTGTAGCGTGTAGAGA	60	[18]

Sox-9	5′-GAG CAG ACG CAC ATC TC 3′-CCT GGG ATT GCC CCG A	60	PRIMER 3

IL-1*β*	5′-GTGGCAATGAGGATGACTTGTT 3′-TGGTGGTCGGAGATTCGTAG	60	PRIMER 3

IL-6	5′-TAGTGAGGAACAAGCCAGAG 3′-GCGCAGAATGAGATGAGTTG	60	PRIMER 3

IL-8	5′-CCAAACCTTTCCACCC 3′-ACTTCTCCACAACCCT	60	PRIMER 3

IL-10	5′-CTTTAAGGGTTACCTGGGTTG 3′-CTTGATGTCTGGGTCTTGG	60	PRIMER 3

TNF-*α*	5′-AGCCCATGTTGTAGCAAACC 3′-ACCTGGGAGTAGATGAGGTA	60	PRIMER 3

VEGF	5′-TGATGATTCTGCCCTCCTC 3′-GCCTTGCCTTGCTGCTC	60	PRIMER 3

FGF-*β*	5′-CGGCTGTACTGCAAAAACGG 3′-TTGTAGCTTGATGTGAGGGTCG	60	PRIMER 3

HGF	5′-ATACTCTTGACCCTCACACC 3′-TGTAGCCTTCTCCTTGACCT	60	PRIMER 3

TGF-*β*1	5′-CAACAATTCCTGGCGATACCT 3′-TAGTGAACCCGTTGATGTCC	60	PRIMER 3

HAS-1	5′-TGGTGCTTCTCTCGCTCTACG 3′-GAACTTGGCAGGCAGGAGG	60	[19]

HAS-2	5′-AAATGGGATGAATTCTTTGTTTATG 3′-GGCGGATGCACAGTAAGGAA	60	[19]

HAS-3	5′-CAGCTGATCCAGGCAATCGT 3′-TGGCTGACCGGATTTCCTC	60	[19]

TIMP-1	5′-CCGACCTCGTCATCAG 3′-GTTGTGGGACCTGTGGAA	60	PRIMER 3

TIMP-3	5′-CCTTGGCTCGGGCTCATC 3′-GGATCACGATGTCGGAGTTG	60	PRIMER 3

IL-4	5′-CAGTTCCACAGGCACAAG 3′-CTGGTTGGCTTCCTTCACA	60	PRIMER 3

IL-13	5′-GCACACTTCTTCTTGGTC 3′-TGAGTCTCTGAACCCTTG	60	PRIMER 3

IL-10	5′-CTTTAAGGGTTACCTGGGTTG 3′-CTTGATGTCTGGGTCTTGG	60	PRIMER 3

MMP-13	5′-TCACGATGGCATTGCT 3′-GCCGGTGTAGGTGTAGA	60	PRIMER 3

∗Primer sequences were obtained from published references and were indicated or designed using PRIMER 3.

**Table 2:** Soluble factor concentrations in fresh (PRP) and frozen PRP 1 h and 7 days after activation. Concentrations are expressed as pg/mL and reported as median values and (interquartile ranges).

Soluble factors	Preparations	Incubation time		*P* value
		1 hour	7 days	
IL-1*β*	PRP	1.015 (0.83–5.41)	70.11 (56.81–233.35)	*P* < 0.01
	Frozen PRP	1.22 (0.72–2.32)	14.43 (1.11–179.20)	NS
	*P* value	NS	NS	

TGF-*β*1	PRP	107861.6 (81652–127793.0)	103553.4 (64935.69–1341400.0)	NS
	Frozen PRP	33849.8 (23339.08–54974.0)	52511.5 (30092.61–201434.0)	NS
	*P* value	*P* < 0.005	*P* < 0.01	

PDGF AB/BB	PRP	27714.68 (18591.50–35850.24)	31670.63 (18617.58–80462.27)	NS
	Frozen PRP	17388.90 (8648.29–29500.03)	6035.78 (4691.41–37053.02)	NS
	*P* value	*P* < 0.01	*P* < 0.01	

VEGF	PRP	157.94 (62.3–238.52)	226.79 (145.82–743.31)	NS
	Frozen PRP	147.78 (9.39–209.94)	204.10 (136.85–632.23)	NS
	*P* value	NS	NS	

HGF	PRP	247.20 (148.75–305.97)	380.89 (370.58–493.17)	*P* < 0.01
	Frozen PRP	253.68 (109.87–283.97)	212.77 (149.44–261.94)	NS
	*P* value	NS	*P* < 0.01	
